# Dysfunctional epigenetic aging of the normal colon and colorectal cancer risk

**DOI:** 10.1186/s13148-019-0801-3

**Published:** 2020-01-03

**Authors:** Ting Wang, Sean K. Maden, Georg E. Luebeck, Christopher I. Li, Polly A. Newcomb, Cornelia M. Ulrich, Ji-Hoon E. Joo, Daniel D. Buchanan, Roger L. Milne, Melissa C. Southey, Kelly T. Carter, Amber R. Willbanks, Yanxin Luo, Ming Yu, William M. Grady

**Affiliations:** 1 Clinical Research Division, Fred Hutchinson Cancer Research Center, D4-100, 1100 Fairview Ave N, Seattle, WA 98109 USA; 2 Computational Biology Program, Oregon Health & Science University, Portland, OR USA; 3 Department of Biomedical Engineering, Oregon Health & Science University, Portland, OR USA; 4 Public Health Sciences Division, Fred Hutchinson Cancer Research Center, Seattle, WA USA; 5 Huntsman Cancer Institute and Department of Population Health Sciences, Salt Lake City, UT USA; 6 Department of Clinical Pathology, Melbourne Medical School, The University of Melbourne, Melbourne, Victoria Australia; 7 Cancer Epidemiology Division, Cancer Council Victoria, Melbourne, Victoria Australia; 8 Centre for Epidemiology and Biostatistics, Melbourne School of Population and Global Health, The University of Melbourne, Melbourne, Victoria Australia; 9 Precision Medicine, School of Clinical Sciences at Monash Health, Monash University, Clayton, Victoria Australia; 10 Department of Colorectal Surgery, The Sixth Affiliated Hospital, Sun Yat-sen University, Guangzhou, Guangdong China; 11 Guangdong Institute of Gastroenterology, Guangdong Provincial Key Laboratory of Colorectal and Pelvic Floor Disease, The Sixth Affiliated Hospital, Sun Yat-sen University, Guangzhou, Guangdong China; 12 Department of Internal Medicine, University of Washington School of Medicine, Seattle, WA USA

**Keywords:** Colorectal cancer, DNA methylation, Epigenetic clock, Biological/epigenetic age, Epigenetic age acceleration

## Abstract

**Background:**

Chronological age is a prominent risk factor for many types of cancers including colorectal cancer (CRC). Yet, the risk of CRC varies substantially between individuals, even within the same age group, which may reflect heterogeneity in biological tissue aging between people. Epigenetic clocks based on DNA methylation are a useful measure of the biological aging process with the potential to serve as a biomarker of an individual’s susceptibility to age-related diseases such as CRC.

**Methods:**

We conducted a genome-wide DNA methylation study on samples of normal colon mucosa (*N* = 334). Subjects were assigned to three cancer risk groups (low, medium, and high) based on their personal adenoma or cancer history. Using previously established epigenetic clocks (Hannum, Horvath, PhenoAge, and EpiTOC), we estimated the biological age of each sample and assessed for epigenetic age acceleration in the samples by regressing the estimated biological age on the individual’s chronological age. We compared the epigenetic age acceleration between different risk groups using a multivariate linear regression model with the adjustment for gender and cell-type fractions for each epigenetic clock. An epigenome-wide association study (EWAS) was performed to identify differential methylation changes associated with CRC risk.

**Results:**

Each epigenetic clock was significantly correlated with the chronological age of the subjects, and the Horvath clock exhibited the strongest correlation in all risk groups (*r* > 0.8, *p* < 1 × 10^−30^). The PhenoAge clock (*p* = 0.0012) revealed epigenetic age deceleration in the high-risk group compared to the low-risk group.

**Conclusions:**

Among the four DNA methylation-based measures of biological age, the Horvath clock is the most accurate for estimating the chronological age of individuals. Individuals with a high risk for CRC have epigenetic age deceleration in their normal colons measured by the PhenoAge clock, which may reflect a dysfunctional epigenetic aging process.

## Background

Colorectal cancer (CRC) is a leading cause of cancer-related death in the USA and arises via a polyp-to-cancer progression sequence. Virtually, all CRCs arise from adenomatous polyps or serrated polyps, although only 5–10% of colon polyps become CRC [1]. Advanced histologic features in the polyp (e.g., villous histology, high-grade dysplasia) and size of the polyp directly correlate with an increased risk of CRC [2]. A precise determination of the factors that mediate polyp initiation and progression would have a major impact on CRC prevention.

At the molecular level, CRC results largely from the progressive accumulation of genetic and epigenetic alterations in colon epithelial cells. DNA methylation alterations commonly occur in adenomas and CRCs and appear to cooperate with gene mutations to mediate field cancerization (also known as “field effect” or “field defect”) in the colon and induce the initiation and progression of adenomas [3–9]. Previous studies evaluating methylation in the normal-appearing colon mucosa have demonstrated an association between DNA methylation of certain cancer-related genes and neoplastic lesions located elsewhere in the colon [10–13]. Methylation of the five genes in the CpG island methylator phenotype (CIMP) panel (*RUNX3*, *SOCS1*, *NEUROG1*, *CACNA1G*, and *IGF2*) was increased in the normal colon of individuals with advanced proximal sessile polyps, the precursor lesion to CIMP cancers [9]. Others have demonstrated a direct correlation between aberrantly methylated *APC*, *DKKI*, *CDKN2A*/*p16*, and *SFRP4* in the apparently normal colon mucosa of cancer patients, and to a lesser extent of polyp patients [14]. Therefore, DNA methylation alterations in the normal colon mucosa could serve as epigenetic markers for colon adenoma and/or CRC risk.

Age is the strongest risk factor for CRC, and advanced age has been associated with an increased risk for advanced polyps and CRC [2, 15]. However, the risk of CRC varies substantially between individuals, even within the same age group, which may reflect heterogeneity in biological tissue aging between people. It has been well appreciated that an individual’s biological age can vary from the chronological age and the biological aging rate differs between individuals [16–19]. These observations have led to efforts to identify accurate markers of biological age. Recently, epigenetic clock CpGs, which are composed of specific sets of methylated CpGs, have been identified as accurate markers of the “true” biological or physiological aging of tissues. For example, Bocklandt et al. generated the first DNAm age estimator using DNA extracted from the saliva [20]. Later, Hannum et al. developed an accurate single-tissue age estimator based on 71 CpGs from peripheral blood leukocyte (PBL) DNA [21]. Horvath constructed the first accurate multi-tissue age estimator based on 353 CpGs using ~ 8000 publicly available microarray samples from over 30 different tissues and cell types collected from children and adults [22]. Levine et al. derived a clock using 513 CpGs to estimate the phenotypic age based on 10 clinical characteristics that associate with the morbidity and mortality risk of individuals (DNAm PhenoAge clock) [23]. Yang et al. built an epigenetic mitotic clock using 385 Polycomb group target (PCGT) promoter CpGs, termed EpiTOC [24]. Interestingly, while EpiTOC, an epigenetic mitotic clock, predicts a universal acceleration in the pan-cancer analysis as well as in normal buccal tissue of smokers [24], the biological age of some cancer types (including CRC) is decelerated [25, 26]. The utility of these clocks in assessing the biological age in normal colon mucosa from people with differing risk of CRC has not been investigated.

In this study, using previously established epigenetic clocks (Hannum, Horvath, PhenoAge, and EpiTOC), we estimated the biological tissue age of the normal colon in individuals within three CRC risk groups. We defined biological age acceleration for each sample by comparing the estimated biological age with the individual’s chronological age, to assess whether accelerated or dysfunctional aging in the colon is associated with an increased CRC risk.

## Methods

### Patient and tissue information

This study included 334 tissue samples of normal colon mucosa collected at the University of Washington Medical Center (Seattle, WA, USA) by endoscopic biopsy from patients undergoing colonoscopies (age 19–85) [27] and by surgical resection from newly diagnosed CRC patients (age 28–89, stages I–IV) [28, 29], following the protocols approved by the Institutional Review Board. To avoid the potentially confounding effects of anatomic location, only the samples from the left colon were included in the study. Genome-wide DNA methylation levels were assessed using the Illumina Infinium HumanMethylation450 (HM450, *N* = 120, completed years 2012–2016) and Infinium MethylationEPIC (EPIC, *N* = 214, completed years 2017–2019) BeadChip arrays.

Risk group assignment was based on the subject’s personal history of adenomas or CRC, which is known to associate with the risk of developing CRC in the future [2]. We defined three risk groups: low, which was based on no concurrent adenomas; medium, which was based on non-advanced adenomas or advanced adenomas (defined as being an adenoma > 1 cm or having tubulovillous histology or high-grade dysplasia); and high, which was based on concurrent CRC. Table 1 summarizes the risk groups and characteristics of the study subjects. We adjusted for the clinical covariates, especially gender and age, and corrected batch effects in our analyses (Additional file 1: Figure S1).

**Table 1:** Study participant characteristics

Characteristic	CRC risk status		
	Low (%) [no concurrent adenomas]	Medium (%) [concurrent adenomas]	High (%) [concurrent cancer]
Total	105 (100.0)	128 (100.0)	101 (100.0)
Gender*			
Female	62 (59.0)	55 (43.0)	39 (38.6)
Male	43 (41.0)	73 (57.0)	62 (61.4)
Age*			
Range (mean)	19–81 (58)	31–85 (63)	28–79 (57)
BMI			
Range (mean)	18–69 (30)	19–48 (30)	19–67 (30)
Not available	5	7	29
Smoking			
Current	13 (12.8)	11 (8.8)	10 (12.5)
Former	24 (23.5)	49 (39.2)	24 (30.0)
Never	65 (63.7)	65 (52.0)	46 (57.5)
Not available	3	3	21
NSAID use			
Yes	44 (43.1)	70 (56.0)	31 (40.3)
No	58 (56.9)	55 (44.0)	46 (59.7)
Not available	3	3	24
Array*			
HM450	48 (45.7)	31 (24.2)	41 (40.6)
EPIC	57 (54.3)	97 (75.8)	60 (59.4)

*NSAID* nonsteroidal anti-inflammatory drug

*p value < 0.05. Chi-square test for category variables, ANOVA *F* test for numerical variables

### DNA extraction and methylation assessment

DNA extraction and bisulfite conversion were performed as described previously [29]. In brief, genomic DNA samples were extracted from the fresh frozen normal colon mucosa tissue samples using the DNeasy Blood and Tissue Kit (Qiagen). Genomic DNA quantification was performed using the Quant-iT PicoGreen DNA assay kit (Life Technologies). DNA (500 ng) from each sample was bisulfite converted using the EZ DNA Methylation Kit (Zymo Research, Irvine, USA). The DNA samples were submitted to the Genomics Core at the Fred Hutchinson Cancer Research Center where they were processed and run on HM450 or EPIC arrays following the manufacturer’s instructions (Illumina, Inc.). The returned raw intensity (IDAT) files were then preprocessed and normalized as described below.

### Methylation array data processing

The raw IDAT files of the two methylation arrays were read into R with the *minfi* package separately [30]; the *combineArrays* function was utilized to combine the two arrays’ data together based on their common CpG sites. Then, the data was preprocessed with background and dye bias correction using the *Noob* method [31], followed by the functional normalization [32]. CpG probes that were SNP-associated, cross-reactive, located on sex chromosomes, and unreliably detected (> 10% of samples with detection *p* value > 0.01), with the exception of the epigenetic clock CpGs, were excluded from the analysis [33–35]. Methylation *β* value for each CpG site in each sample was calculated as *M*/(*M* + *U* + *α*), where *M* and *U* represent methylated and unmethylated signal intensities at the CpG site, respectively, and *α* is an arbitrary offset (usually 100) intended to stabilize *β* values where fluorescent intensities are low. NA values, if existed after the QC filtering, were imputed as the means of all non-NA values of the corresponding CpGs. The *β* values were transformed into *M* values as log_2_(*β*/(1 – *β*)), and the batch effects were removed based on the *M* values using the *Combat* approach [36].

Of note, 64% of the samples were run on the EPIC array platform, while the rest were run on the HM450 array (Table 1 and Additional file 1: Figure S2). In our study, we first analyzed the data of the two array platforms separately and found comparable results with regard to the determined epigenetic age and acceleration and CRC risk; we also performed an inverse variance-based meta-analysis [37] to combine the testing statistics and *p* values of the two datasets and confirmed the results of using the combined dataset are similar to those obtained from the separate datasets (see results in the “Discussion” section). Therefore, we finally combined the EPIC and HM450 datasets to increase the sample size and gain more statistical power for the studies we conducted.

### Estimation of cell-type fractions

Cell-type heterogeneity may cause somatic DNA methylation variation between tissue samples and may be an important confounder in the study of DNAm and epigenetic age alterations in association with CRC risk in the normal colon tissues [18]. Therefore, we used *EpiDISH* [38], a reference-based algorithm for the inference of cell-type proportions in cell mixture samples, to estimate the fractions of epithelial cells, fibroblasts, and total immune cells in our samples.

### Calculation of epigenetic age

The epigenetic ages of each sample were estimated using 4 popular epigenetic clocks, which were the Hannum clock, which relies on 71 CpGs identified in blood DNA samples [21]; the Horvath clock, which relies on 353 CpGs and is based on the analysis of DNA methylation from multiple tissue types [22]; the PhenoAge clock, which is based on 513 CpGs derived to measure phenotypic aging [23]; and the EpiTOC clock, which is derived from the analysis of 385 Polycomb group target promoter CpGs [24]. Note these epigenetic clocks were developed using data from the HM450 array. Although the EPIC array lacks some of the CpGs in the Hannum (6), Horvath (19), and EpiTOC (31) clocks due to the differences in the array design between the HM450 and EPIC arrays (Additional file 1: Figure S3), McEwen et al. have demonstrated that the missing clock CpGs on the EPIC array do not substantially affect the accuracy of the Hannum or Horvath age determination [39]. To verify this observation for all 4 clocks, we performed a sensitivity analysis on our HM450 data. We selected the common clock CpGs on both arrays to calculate the epigenetic ages for the HM450 samples and compared these results with their epigenetic ages derived from using all clock CpGs (see results in the “Discussion” section).

A linear regression model was used to describe the relationship between the epigenetic age and chronological age at the time of tissue collection. The deviation between epigenetic age and chronological age, also known as epigenetic age acceleration, was calculated for every sample based on the residuals of regressing the epigenetic ages on the chronological ages of all the samples, as described by McEwen et al. [39].

### Statistical methods

The correlation between epigenetic ages and chronological ages of the samples was calculated with the Pearson correlation coefficient. To investigate the change of this association between the different CRC risk groups, a linear regression model with interaction effect was adopted by considering the chronological age as a linear predictor and the risk status as a categorical predictor. To test the association of epigenetic clock with cancer risk, a multivariate linear regression was applied with the epigenetic age acceleration as the dependent variable and the cancer risk as the independent variable with adjustment for other covariates, such as gender and cell-type fractions. When considering other clinical variables that might be able to affect DNA methylation or aging, such as the BMI, smoking, and NSAID use (Table 1), a subset of 293 samples that had no missing data of these variables were analyzed. The Fligner-Killeen test was used to test the homogeneity of variances between different CRC risk groups.

### Epigenome-wide association study of CRC risk

We implemented the Surrogate Variable Analysis (*sva*) [40] on the DNAm data (*M* values) by setting a null model matrix (mod0 ~ age + gender) and a full model matrix (mod ~ risk + age + gender) to estimate the surrogate variables (SVs) that represented other latent confounding factors. Then, an epigenome-wide association study (EWAS) was performed to identify DNAm changes between different CRC risk groups using a multivariate linear regression model, where DNAm level of a CpG was the outcome, CRC risk status was the independent variable of interest, and age, gender, and SVs were the adjustment variables. The output included effect size (i.e., *M* value mean difference) and *p* value for each CpG. The false discovery rate (*FDR*)-adjusted *p* values were calculated for the multiple testing adjustment. FDR < 0.01 was used to determine the differentially methylated CpGs between different risk groups. One-way Fisher’s exact test was performed on a two-by-two contingency table, which contained the numbers of differentially methylated CpGs (from the EWAS analysis) in a clock and in a whole array as well as the numbers of total CpGs in the corresponding clock and array to test if the clock was enriched with more differential CpGs. Gene Ontology (GO) functional annotation for the genes close to the differentially methylated clock CpGs was analyzed using the online Database for Annotation, Visualization and Integrated Discovery (DAVID) v6.8 [41].

## Results

### Assessment of epigenetic clocks in normal colon mucosa

**Fig. 1:**
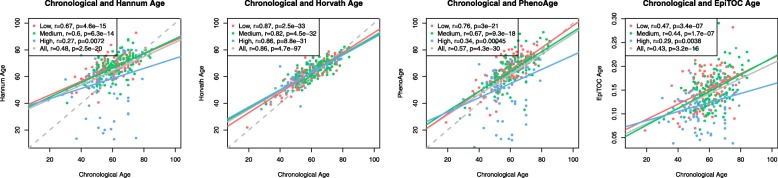
Correlation of four epigenetic age estimates (Hannum, Horvath, PhenoAge, and EpiTOC) in the normal colon with the chronological age of the individuals providing the normal colon samples. Different colors represent different groups based on the CRC risk status

To study epigenetic aging in the normal colon and its correlation with the risk for developing colorectal cancer, we conducted a genome-wide DNA methylation study on normal colon mucosa samples collected from patients assigned to the low, medium, and high CRC risk groups (*N* = 105, 128, and 101, respectively, see Table 1 for subject information). We calculated the Pearson correlation coefficient between the epigenetic age and chronological age for subjects in each risk group and for the combined set of samples. We found that the Horvath clock had the strongest and most significant correlation with the chronological age in all of the groups (*r* > 0.8, *p* < 1 × 10^−30^), while the Hannum, PhenoAge, and EpiTOC clocks showed weaker correlations with chronological age, particularly in the high-risk group (Fig. 1). The linear regression with interaction effect did not reveal significant changes in the association of epigenetic age with chronological age between the risk groups (*p* > 0.05 in all tests). Our observations demonstrate that the Horvath clock is the most accurate clock for predicting the chronological age in the normal colon and that the epigenetic ages derived from the Hannum, PhenoAge, and EpiTOC clocks diverge from the chronological age of the samples.

### Association of epigenetic age with colorectal cancer risk

Next, we analyzed the association between the epigenetic ages of the samples and their CRC risk status to determine whether the biological tissue age of the colon was associated with an increased risk of developing CRC. We initially assessed the epigenetic ages of the samples using the four different epigenetic clocks and found an older mean age in the medium-risk group for the Horvath clock but a younger mean age in the high-risk group for the Hannum, PhenoAge, and EpiTOC clocks compared to the low-risk group (Additional file 1: Figure S4). In addition, the analysis using the Fligner-Killeen test revealed that the Hannum (*p* = 7.5 × 10^−4^) and PhenoAge (*p* = 0.047) epigenetic ages of the high-risk samples had a significantly larger amount of variances compared to the low-risk samples. To adjust for the bias due to individual chronological age, we assessed epigenetic aging using a popular measure named epigenetic age acceleration, which was obtained from the residuals of regressing epigenetic ages of all the samples onto their chronological ages [39].

Cell-type heterogeneity may cause somatic DNA methylation variation between different groups of colon samples and may be an important confounder affecting epigenetic clocks in association with CRC risk [18]. Therefore, we estimated the fractions of epithelial cells, fibroblasts, and total immune cells within each sample using the *EpiDISH* algorithm [38]. We found that cell-type fractions were highly correlated with top PCs of the DNAm data (Additional file 1: Figure S5A), indicating cell-type heterogeneity had a considerable influence on the DNAm results of the samples. The percentage of fibroblasts was significantly higher (*p* = 2.6 × 10^−11^), and the percentage of total immune cells was significantly lower (*p* = 2.8 × 10^−9^) in the high-risk group compared to the low-risk group (Additional file 1: Figure S5B). We also found that the estimated cell-type fractions were correlated with the Hannum, PhenoAge, and EpiTOC age estimates but were not correlated with the Horvath age estimate (Additional file 1: Figure S5C), perhaps because the Horvath clock was built on large-scale multi-tissue data and hence can adjust for the influence of cell-type heterogeneity intrinsically, while the other three clocks are more sensitive to changes in cell-type composition.

**Fig. 2:**
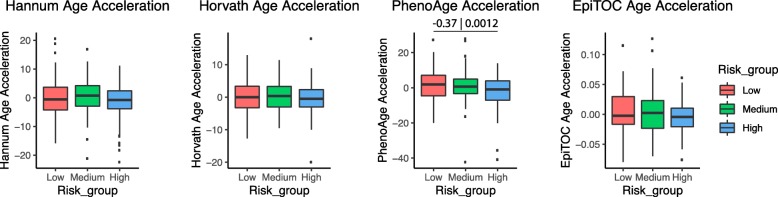
Distribution of epigenetic age acceleration in the three CRC risk groups. The *y*-axis shows the epigenetic age acceleration after adjusting for gender and cell-type fractions (i.e., residual of regressing the epigenetic age acceleration on gender and cell-type fractions). Standardized effect size (i.e., Cohen’s *d*) and *p* value for the significant association (*p* value < 0.01) is shown above the corresponding line

By taking the potential confounders into consideration, we used a multivariate linear regression with the adjustment for the gender and the estimated cell-type fractions to test the difference of epigenetic age acceleration between the CRC risk groups. We observed significant deceleration of PhenoAge (*p* = 0.0012) in the high-risk samples compared to the low-risk samples (Fig. 2). A similar phenomenon was also observed after additionally adjusting for other relevant clinical variables, such as BMI, smoking, and NSAID use, in the regression model using a subset of 293 samples that had sufficient clinical annotation for these variables (Additional file 1: Figure S6).

To validate our results and considering the lack of datasets from genome-wide DNAm analysis of normal colons from healthy individuals, we generated 2 HM450 datasets by (1) combing the raw IDAT files of our low-risk normal colon samples (*N* = 48, UWAS-Low) with the TCGA-COAD adjacent normal left colon samples (*N* = 9, TCGA-High) and (2) combing the raw IDAT files of our 48 low-risk normal colon samples with normal left colon samples from patients with CRC from the Australian Melbourne Collaborative Cohort Study (*N* = 14, MCCS-High) [42]. We repeated all the analyses using these datasets and observed epigenetic age deceleration in the TCGA-High group for the Hannum, PhenoAge, and EpiTOC clocks and in the MCCS-High group for the Hannum and EpiTOC clocks (Additional file 1: Figure S7, *p* < 0.05).

We wish to note that it might be also interesting to estimate the epigenetic age of matched cancer tissues from the high-risk group patients; hence, we assessed the CRC samples from a subset of the people with cancer (*N* = 13). We combined all the normal colon and CRC samples together and assigned them into four groups (low, medium, high, and CRC). We determined the epigenetic age in these samples and found that the Horvath clock was significantly decelerated while the PhenoAge and EpiTOC clocks were significantly accelerated in the CRC samples (Additional file 1: Figure S8).

### DNA methylation changes in association with CRC risk and impact on epigenetic clocks

In light of our observation of deceleration of the epigenetic clocks in the high-risk normal colon samples, we next assessed for epigenome-wide methylation changes in association with CRC risk status of the samples in order to determine if the risk-associated methylation changes in the normal colon were skewing the performance of the epigenetic clocks. We performed an EWAS analysis on the methylation data of all 334 samples to identify genome-wide DNA methylation changes that were associated with cancer risk by applying a multivariate linear regression to each CpG. Using a significance threshold of FDR < 0.01, we identified 14,947 differentially methylated CpGs in the high-risk group compared to the low-risk group (see Manhattan, QQ, and histogram plots of EWAS *p* values in Additional file 1: Figure S9A). We noticed that 5 of the Hannum clock CpGs, 18 of the Horvath clock CpGs, 20 of the PhenoAge clock CpGs, and 20 of the EpiTOC clock CpGs were differentially methylated in the high-risk group vs. the low-risk group (see volcano plots in Additional file 1: Figure S9B). None of the four clocks was significantly enriched with differentially methylated CpGs (Fisher’s exact test *p* value > 0.01). Gene Ontology (GO) functional annotation for the genes close to these differentially methylated clock CpGs indicated that they were significantly relevant to the biological process of “cardiac cell fate determination” (*p* value < 0.01). We further investigated the relationship between the methylation mean differences of the clock CpGs in the high-risk group derived from the EWAS and their coefficients (or weights) in the weighted sum-based epigenetic clock models (i.e., *y* = *βX*). We wish to note the EpiTOC clock is an average methylation model, where each clock CpG has the same coefficient that is 1/385. We multiplied the coefficient of each clock CpG by its EWAS mean difference to quantify its overall mean difference in terms of the epigenetic clock (see the scatter plots in Additional file 1: Figure S9C). Although some CpGs in the PhenoAge clock were significantly hypermethylated in the high-risk group, their negative clock coefficients made them contribute to the observed age deceleration. In contrast, the EpiTOC clock was directly affected by methylation changes of the clock CpGs.

## Discussion

Aging is associated with a variety of diseases, including cancer. The risk for cancer and other age-related diseases varies dramatically between individuals. Furthermore, it appears that some people age prematurely at a biological level and are consequently at increased risk for age-related diseases, such as heart disease and dementia [43–45]. Thus, there is an intense interest in identifying accurate markers for the biological aging process. Recently, the epigenetic clock and epigenetic/biological aging have been shown to predict a variety of age-related physiologic decline processes and age-related diseases, and individuals with these diseases often have an acceleration of their epigenetic clocks [18]. In this study, we have assessed the association between epigenetic age and risk for developing colorectal cancer using a variety of established epigenetic clocks, the Hannum [21], Horvath [22], PhenoAge [23], and EpiTOC [24] clocks. We found that the individuals with the highest risk for CRC had a significant deceleration of PhenoAge in their normal colons compared to the normal colons of low-risk individuals. We also found that the Horvath clock is the most accurate clock for estimating the chronological age of normal colon samples.

Colorectal cancer is primarily a disease of the elderly and is believed to arise in large part secondary to age-related changes in the colon. A variety of age-mediated cellular and molecular mechanisms have been proposed to induce a tendency for tissues to transform into cancer. These mechanisms include cellular senescence, the accumulation of mutations in stem cells, long-term exposure to oxidative exposures, and increased mutation rates [46–49], among others. More recently, the accumulation of epigenetic alterations in aged tissues has been proposed as a cancer-causing molecular mechanism in the colon. One example is the age-related DNA methylation affects genes in the key Wnt signaling pathway in the normal colon crypts [13, 14]. Although speculative, our results raise the possibility that deregulation of the epigenetic clocks as reflected in the decelerated aging we observe in the normal colon of people with CRC, rather than strict acceleration, may be occurring in the colon of people at risk for developing CRC. Similarly, prior studies have shown epigenetic age deceleration in the subsets of breast cancers and colorectal cancers [22, 26, 50]. These observations suggest that the process of carcinogenesis may involve disruption, rather than only acceleration, of epigenomic maintenance systems that can result in deceleration of epigenetic clocks in cancers and cancer-prone tissues. Our results are consistent with this possibility. Recently, Marwitz et al. observed epigenetic age deceleration in squamous cell carcinoma of the lungs and that stem cell-related gene expression was increased in these cancers [51]. This raises the possibility that epigenetic age deceleration in the high-risk normal colons may reflect expansions of the stem cell pool, which could increase CRC risk. Other possible explanations for our findings include that the epigenetic clocks are not trained specifically in colon tissue and do not accurately measure epigenetic aging in the colon or that the epigenetic aging process is altered in colon cancerization in a way that invalidates the current epigenetic clocks in use.

Our observations indicate that different epigenetic clocks may essentially assess different aspects of aging. The Horvath clock has strong correlation with the chronological age but no association with the CRC risk; it appears to result from an intrinsic aging process that is not affected substantially by cell type/composition, cell proliferation, or environmental factors; while the Hannum, PhenoAge, and EpiTOC clocks exhibit differences between the CRC risk groups, they may better measure not only the internal clock mechanism of cell division but also exposure to phenotypic epi-mutagens during cancer progression. We wish to note that while our manuscript was under review, Lu et al. have published an epigenetic predictor of lifespan and mortality, named DNAm GrimAge, in 2019 [52]. We have assessed this estimator using our normal colon dataset and observed that the GrimAge is significantly correlated with individual chronological age and that GrimAge and GrimAge acceleration are not associated with CRC risk (Additional file 1: Figure S10). Based on our findings, we suggest that the relevant epigenetic clock to study the processes related to CRC formation is not clear at this time.

It is noteworthy that our study has certain limitations that may have affected our results. Among the various extrinsic and intrinsic risk factors, the unique tissue environment of the colon, which includes intimate interactions with the gut microbiome and diet digestion products, may result in organ-specific effects on cancer risk and affect the epigenetic clocks in a tissue-specific way. If this is true, there may be a need to develop a colon-specific clock. In addition, it is likely that there is heterogeneity in the factors affecting CRC risk among the subjects in each risk group, which may limit our ability to detect a difference based on our sample size. We also wish to note that in our study, we combine 214 EPIC array samples and 120 HM450 array samples (Additional file 1: Figure S2). To determine if the use of the 2 different methylation array platforms may have adversely affected our results due to missing clock CpGs on the EPIC array (Additional file 1: Figure S3), we performed a sensitivity analysis on our HM450 array data by selecting the common clock CpGs on both arrays to recalculate the 4 epigenetic ages and accelerations for the HM450 samples. Consistent with McEwen et al. [39], the missing CpGs did not significantly affect the accuracy of the epigenetic age determination in our samples. We observed nearly identical associations of the epigenetic ages with chronological age as well as with cancer risk (Additional file 1: Figure S11) to those of using all available CpGs on the HM450 array (Additional file 1: Figure S12). Furthermore, when we separately analyzed the data of the 2 arrays, we obtained comparable results (Additional file 1: Figures S12–S13). We used an inverse variance-based meta-analysis [37] to combine the testing effect sizes and *p* values from the 2 datasets and obtained similar results (*p* = 0.006 and 0.010 for the PhenoAge and EpiTOC clocks, respectively) to those from the combined dataset. Therefore, we have demonstrated the feasibility and rationality of combining the HM450 and EPIC data and using the common clock CpGs to estimate the epigenetic ages of the samples and study their associations with individual chronological age and CRC risk. We also wish to note that although we have replicated the observation of epigenetic age deceleration in high-risk normal colons in 2 validation datasets, the 2 datasets are not completely independent of our own dataset and that the results could be subject to the confounding effect of the cohort/batch.

## Conclusions

We have investigated four established epigenetic clocks and their associations with the risk of developing CRC. Our results indicate that (1) the Horvath clock is the most accurate for estimating the chronological age of individuals, (2) individuals at medium CRC risk have no evidence of biological tissue age acceleration or deregulation, and (3) individuals at high CRC risk have deceleration of PhenoAge in their normal colons. Our results suggest the epigenetic aging process is deregulated in the normal colon of people at high risk for CRC, but the mechanisms driving the deregulation remain to be defined.

## Supplementary information

**Figure S1.** Distributions of gender, age, batch and CRC risk of the studied samples. **Figure S2.** Distributions of gender and age in three CRC risk groups in the EPIC and HM450 array datasets. **Figure S3.** Venn diagrams of epigenetic clock CpGs on the HM450 and EPIC methylation arrays. **Figure S4.** Distribution of epigenetic age in three CRC risk groups. **Figure S5.** Association of the estimated cell-type fractions with DNAm data, individual CRC risk and chronological/epigenetic ages. **Figure S6.** Distribution of epigenetic age acceleration in three CRC risk groups, with the adjustment for gender, cell-type fractions, BMI, smoking and NSAID use of subjects. **Figure S7.** Replication results of comparing epigenetic age acceleration between the low and high risk normal colons using two validation datasets. **Figure S8.** Analysis of the combined dataset of normal colon and CRC samples. **Figure S9.** EWAS results of CRC risk. **Figure S10.** Association of DNAm GrimAge in the normal colon with individual chronological age and CRC risk. **Figure S11.** Sensitivity analysis of the HM450 array dataset using the common clock CpGs on both HM450 and EPIC arrays. **Figure S12.** Epigenetic age estimates of the samples in the HM450 array dataset. **Figure S13.** Epigenetic age estimates of the samples in the EPIC array dataset.

## Data Availability

The raw methylation IDAT files and processed data are available online at the GEO website (Accession number is GSE132804).

## Abbreviations

CpG: Cytosine-phosphate-guanine
CRC: Colorectal cancer
EWAS: Epigenome-wide association study
FDR: False discovery rate
