# Supplementary material for: Dysfunctional epigenetic aging of the normal colon and colorectal cancer risk
Source: Clin Epigenetics. 2020 Jan 3;12:5. doi: 10.1186/s13148-019-0801-3 (PMC6942339; doi:10.1186/s13148-019-0801-3)
Supplement: Supplementary file 1 — Figure S1. Distributions of gender, age, batch and CRC risk of the studied samples. Figure S2. Distributions of gender and age in three CRC risk groups in the EPIC and HM450 array datasets. Figure S3. Venn diagrams of epigenetic clock CpGs on the HM450 and EPIC methylation arrays. Figure S4. Distribution of epigenetic age in three CRC risk groups. Figure S5. Association of the estimated cell-type fractions with DNAm data, individual CRC risk and chronological/epigenetic ages. Figure S6. Distribution of epigenetic age acceleration in three CRC risk groups, with the adjustment for gender, cell-type fractions, BMI, smoking and NSAID use of subjects. Figure S7. Replication results of comparing epigenetic age acceleration between the low and high risk normal colons using two validation datasets. Figure S8. Analysis of the combined dataset of normal colon and CRC samples. Figure S9. EWAS results of CRC risk. Figure S10. Association of DNAm GrimAge in the normal colon with individual chronological age and CRC risk. Figure S11. Sensitivity analysis of the HM450 array dataset using the common clock CpGs on both HM450 and EPIC arrays. Figure S12. Epigenetic age estimates of the samples in the HM450 array dataset. Figure S13. Epigenetic age estimates of the samples in the EPIC array dataset. [file 13148_2019_801_MOESM1_ESM.docx]

**Figure S1** | (A) Distribution of individual gender in different risk groups of samples, the difference is tested by Chi-Square test. (B) Distribution of individual age in different risk groups of samples, tested by ANOVA F-test. (C) PCA plot of DNAm data indicates batch effects due to different array platforms and running dates, which are corrected using the *Combat* batch effect removal approach. (D) PCA plot of DNAm data with samples marked in different colors of CRC risk.

**Figure S2** | Distributions of gender and age in three CRC risk groups in the EPIC (A) and HM450 (B) array datasets. The difference of gender among the three groups is tested by Chi-Square test, and the difference of age among the three groups is tested by ANOVA F-test.

**Figure S3** | Venn diagrams showing shared and distinct epigenetic clock CpGs of each clock on the HM450 and EPIC methylation arrays.


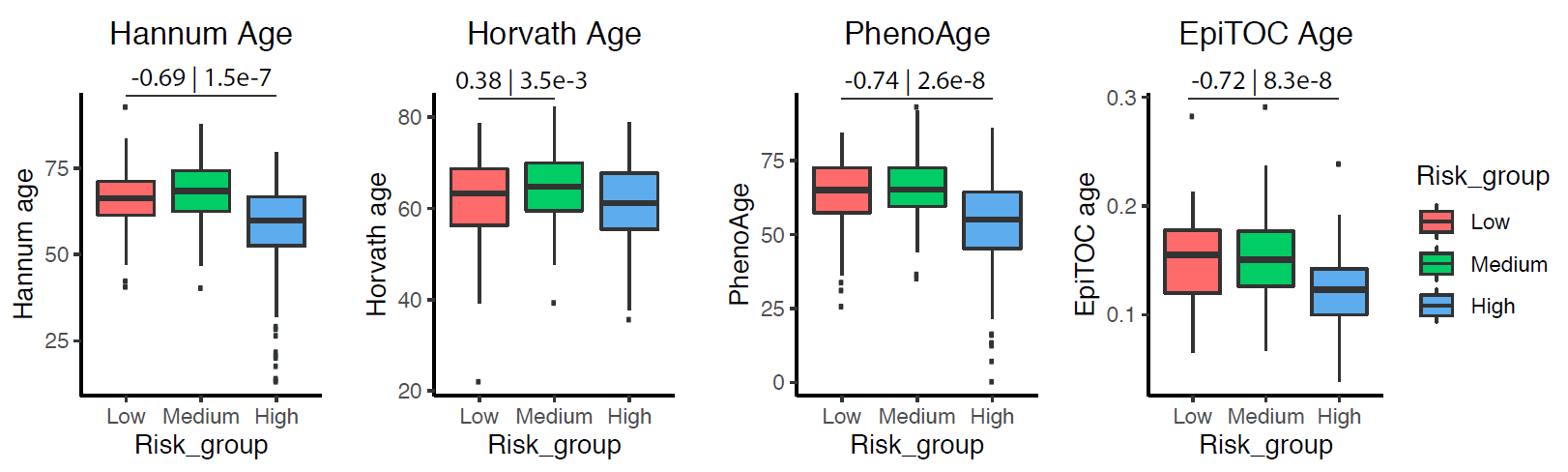


**Figure S4** | Distribution of epigenetic age in three CRC risk groups. Standardized effect size (i.e. Cohen's *d*) | *p*-value for the significant associations (*P*-value < 0.01) are shown above the corresponding lines.

**Figure S5** | Estimation of cell-type (epithelial cells, fibroblasts, and total immune cells) fractions in the studied normal colon samples. (A) Heatmap of correlation between cell-type fractions and top 10 PCs of DNAm data. (B) Distribution of cell-type fractions in three CRC risk groups, tested by ANOVA. (C) Heatmap of correlation between cell-type fractions and individual chronological age and epigenetic age estimates.


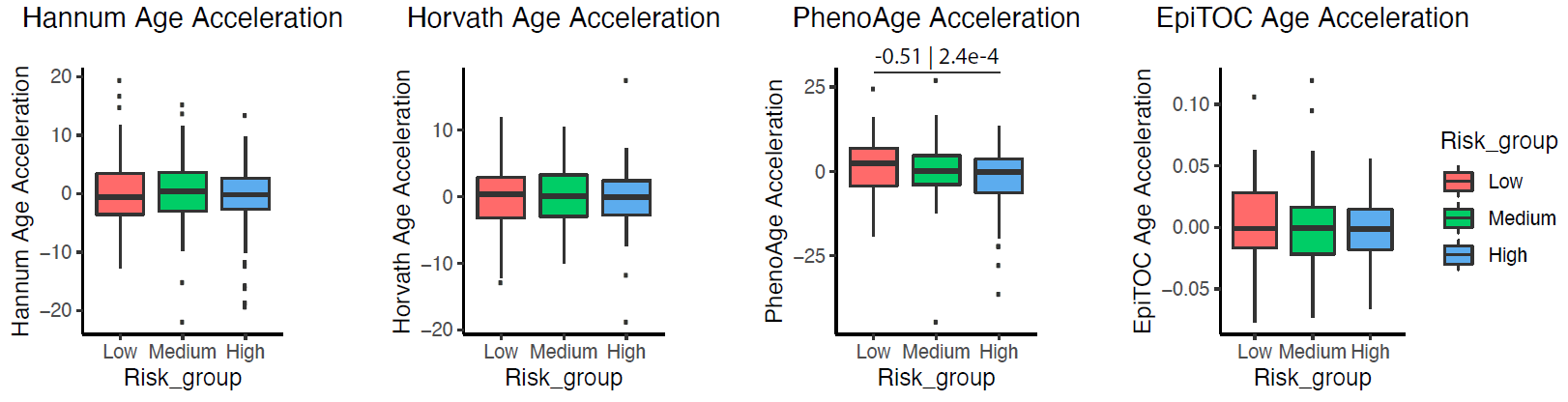


**Figure S6** | Distribution of epigenetic age acceleration in three CRC risk groups, with the adjustment for gender, cell-type fractions, BMI, smoking and NSAID use of subjects. The y-axis shows the epigenetic age acceleration after adjusting for all these covariates (i.e. residual of regressing the epigenetic age acceleration on these covariates). Standardized effect size (i.e. Cohen's *d*) | *p*-value for the significant associations (*P*-value < 0.01) are shown above the corresponding lines.

**Figure S7** | Replication results of comparing epigenetic age acceleration between the low and high risk normal colons using two validation datasets that are generated by: (A) combing the raw IDAT files of our low risk normal colon samples (N=48, UWAS-Low) with the TCGA-COAD adjacent normal left colon samples (N=9, TCGA-High); (B) combing the raw IDAT files of our 48 low risk normal colon samples with the MCCS normal left colon samples from patients with CRC (N=14, MCCS-High). The y-axis shows the epigenetic age acceleration after adjusting for gender and cell-type fractions. Standardized effect size (i.e. Cohen's *d*) | *p*-value are shown above the comparison lines.

**Figure S8** | Analysis of the combined dataset of normal colon and CRC samples. (A) Distribution of gender, age and cell-type fractions in different groups of normal colon and CRC samples. (B) Correlation of epigenetic ages in normal colon and CRC samples with individual chronological age. (C) Distribution of epigenetic age acceleration in different groups of normal colon and CRC samples. The y-axis shows the epigenetic age acceleration after adjusting for gender and cell-type fractions. Standardized effect size (i.e. Cohen's *d*) | *p*-value for the significant associations (*P*-value < 0.05) are shown above the corresponding lines.


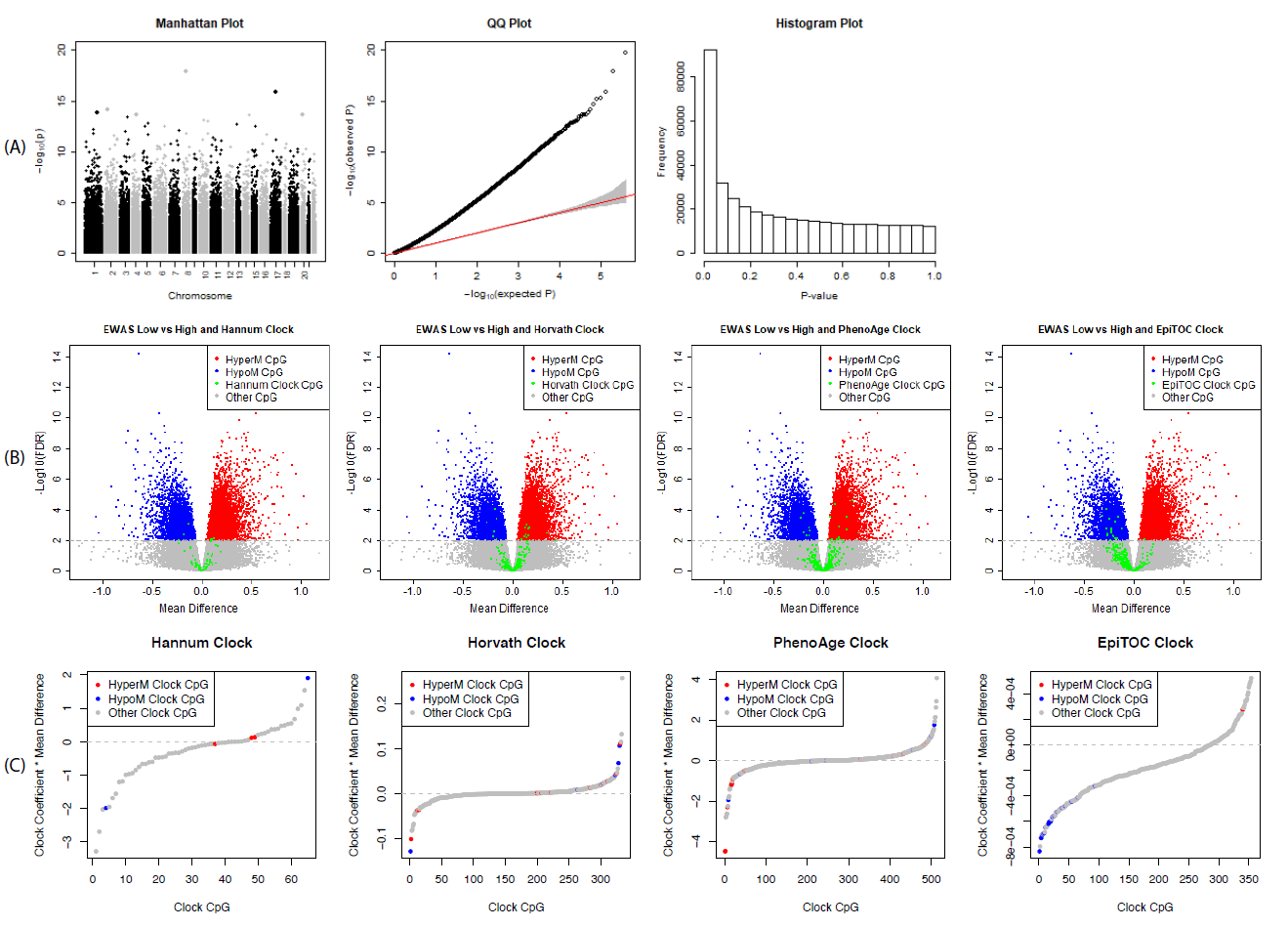


**Figure S9** | (A) The Manhattan, QQ and Histogram plots show distributions of EWAS *p*-values. (B) The volcano plots show EWAS *FDR* and methylation *M*-value mean difference for all CpGs including the clock CpGs. (C) The scatter plots show results of multiplying the coefficient of each clock CpG by its methylation mean difference to quantify its overall mean difference in terms of the epigenetic clock.

**Figure S10** | Association of DNAm GrimAge in the normal colon with individual chronological age and CRC risk.

**Figure S11** | Sensitivity analysis of the HM450 array dataset using the common clock CpGs on both HM450 and EPIC arrays. (A) The scatter plots show correlation of epigenetic age estimates with individual chronological age. (B) The box plots show distribution of epigenetic age acceleration in three CRC risk groups. The y-axis shows the epigenetic age acceleration after adjusting for gender and cell-type fractions. All the results are nearly identical to those of using all the CpGs on the HM450 array (Figure S12), indicating that the missing clock CpGs on the EPIC array do not significantly affect the epigenetic age determination.

**Figure S12** | Epigenetic age estimates of the samples in the HM450 array dataset. (A) The scatter plots show correlation of epigenetic age estimates with individual chronological age. (B) The box plots show distribution of epigenetic age acceleration in three CRC risk groups. The y-axis shows the epigenetic age acceleration after adjusting for gender and cell-type fractions.

**Figure S13** | Epigenetic age estimates of the samples in the EPIC array dataset. (A) The scatter plots show correlation of epigenetic age estimates with individual chronological age. (B) The box plots show distribution of epigenetic age acceleration in three CRC risk groups. The y-axis shows the epigenetic age acceleration after adjusting for gender and cell-type fractions.
